# Pure squamous cell carcinoma of the pancreas: a rare and challenging diagnosis (case report)

**DOI:** 10.11604/pamj.2023.45.4.29484

**Published:** 2023-05-03

**Authors:** Fatima Belabbes, Mohamed Bouziane, Wafaa Kaikani, Abderrahmane Al Bouzidi, Youssef Bennani

**Affiliations:** 1 Department of Gastroenterology and Proctology, Mohammed VI University of Health Sciences, Cheich-Khalifa International University Hospital, Casablanca, Morocco,; 2 Department of Surgery, Mohammed VI University of Health Sciences, Cheikh-Khalifa International University Hospital, Casablanca, Morocco,; 3 Department of Oncology, Mohammed VI University of Health Sciences, Cheikh-Khalifa International University Hospital, Casablanca, Morocco,; 4 Department of Anapathology, Mohammed VI University of Health Sciences, Cheikh-Khalifa International University Hospital, Casablanca, Morocco

**Keywords:** Squamous cell carcinoma, endoscopic ultrasonography, pancreas, case report

## Abstract

Squamous cell carcinoma (SCC) of the pancreas is a rare exocrine ductal tumour with unknown pathophysiology and poor treatment options. We present a case of SCC in a 59-year-old male patient who presented with epigastric pain, vomiting, anorexia, asthenia, and weight loss. Abdomino-pelvic CT revealed a pancreatic mass with adenopathy satellites. Surgical biopsies were negative, and a trans-duodenal gastric endoscopy showed suspicious lymphadenopathies and a hypoechoic lesion invading the portal vein. An anapathological study confirmed SCC at the site of intense necrotic and inflammatory changes. The patient received radio-chemotherapy, but ultimately developed peritoneal carcinoma.

## Introduction

Squamous cell carcinoma of the pancreas is a rare malignant exocrine ductal tumour with an incidence rate of 0.5-5% of all malignant tumours of the pancreas [1]. Its pathophysiology remains unknown, and the histogenesis is uncertain, making treatment options limited. Prognosis is poor, and the literature on this topic is limited. We report a case of SCC in a 59-year-old male patient.

## Patient and observation

**Patient information:** the patient, a 59-year-old male, had a history of hemorrhoids complicated by infection (Fournier's gangrene) requiring a clean left iliac colostomy. He presented with predominantly epigastric abdominal pain accompanied by vomiting, anorexia, asthenia, and weight loss of 5kg in 20 days.

**Clinical findings:** abdominal exam revealed an epigastric impasto. No ascites, palpable lymphadenopathy or troisier ganglion were found. Nothing unusual was found during the digital rectal examination.

**Figure 1:**
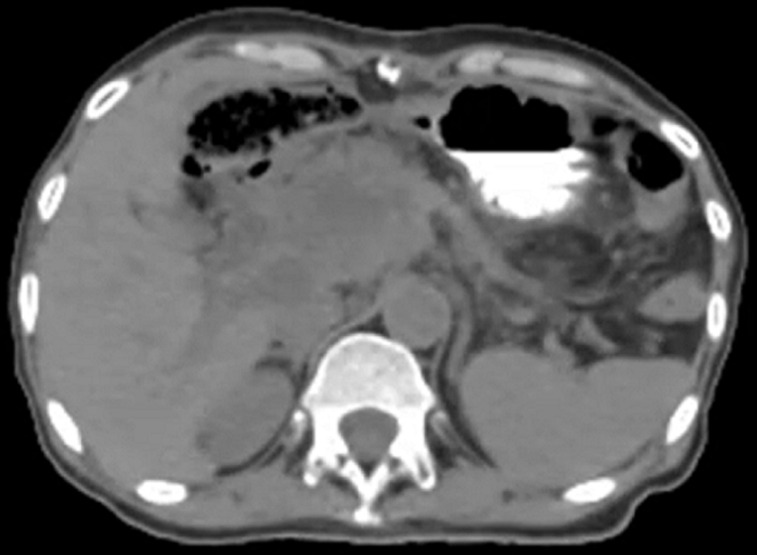
abdominal CT scan showing a hypodense pancreatic mass

**Figure 2:**
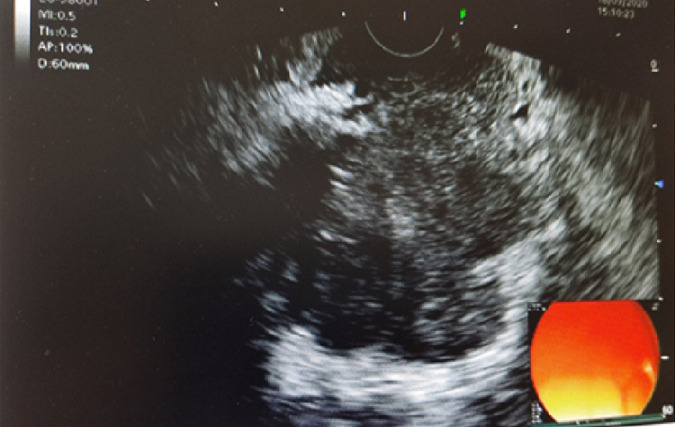
endoscopy showing a hypoechoic mass with invasion of the portal trunk

**Figure 3:**
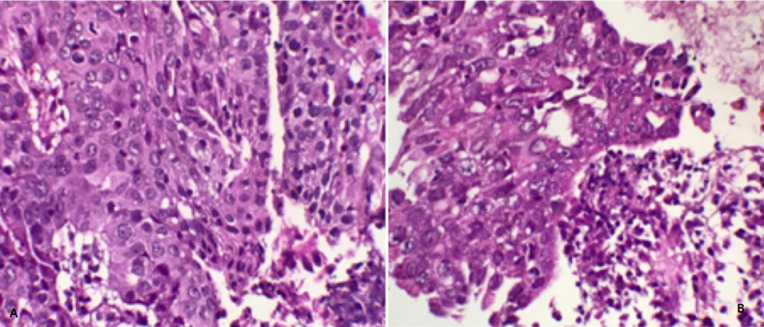
tumor cells are large and cohesive (HE, Gx400)

**Diagnostic assessment:** an abdomino-pelvic CT showed a pancreatic mass with adenopathy satellites (Figure 1). Tumour markers reached a level of CA19-9: 1286.6 IU/ml and C - reactive protein was increased by a level of 30.50 mg/l. A trans-duodenal gastric endoscopy showed suspicious lymphadenopathies and a hypoechoic lesion invading the portal vein (Figure 2). An anapathological study confirmed SCC at the site of intense necrotic and inflammatory changes (Figure 3).

**Therapeutic intervention:** the patient received radio-chemotherapy without cisplatin due to his general condition.

**Follow-up and outcomes:** the patient ultimately developed peritoneal carcinoma.

**Informed consent:** the patient provided informed consent.

## Discussion

Epidermoid carcinoma of the pancreas is a rare form of exocrine pancreatic cancer. Histologically, it is a rare epithelial tumour of the exocrine pancreas as there are typically no natural squamous cells in the pancreas [2]. Squamous cell carcinoma, compared to adenocarcinoma, which is the most common subtype of pancreatic carcinoma, has the same epidemiological criteria but a lower survival rate.

The pathophysiology of squamous cell carcinoma of the pancreas is still unknown, as the pancreas does not usually contain squamous cells [2]. Several theories have been proposed to explain the origin of these tumours, including malignant transformation of squamous metaplasia following chronic inflammation, differentiation from a primitive bipotential cell, or squamous transformation of pre-existing adenocarcinoma [3].

Diagnosing and treating squamous cell carcinoma of the pancreas presents challenges in clinical practice. The clinical presentation and course of the disease are similar to those of adenocarcinoma. Radiologically, on the abdominal CT scan, peripheral contrast enhancement is typically observed in the lesion after the injection of contrast product. Epidermoid carcinomas are usually large in size, with lymph node metastasis [2,3]. A tissue sample is necessary for diagnosis and the orientation of subsequent management, usually by endoscopic ultrasound with fine needle aspiration.

Various therapeutic methods have been used to treat pancreatic SCC, including surgical resection, chemotherapy regimens, and radiation therapy, but none have been proven effective [3]. No optimal treatment has been validated according to the tumour stage, although surgical resection has been the only curative option. Radiation therapy or chemotherapy has been performed for the remaining cases [4]. The optimal treatment regimen remains unknown [5], although radiation therapy, platinum-based regimens, gemcitabine, and 5-FU have all been reported to have favorable results [6]. Resectability and low/intermediate grades are favorable prognostic factors [7]. For patients with unresectable diseases, palliative treatment cannot decrease mortality [8].

## Conclusion

Epidermoid carcinoma of the pancreas is a rare tumour that should be considered in the differential diagnosis of pancreatic masses. EUS-FNA is a valuable tool for diagnosis, and surgical resection remains the only curative treatment option. No optimal treatment strategy has been established for inoperable pancreatic squamous cell carcinoma (SCC). Further studies are needed to establish effective adjuvant therapies and improve the prognosis of this rare disease.
